# Safety and efficacy of the CELT ACD femoral arteriotomy closure device in the office-based laboratory

**DOI:** 10.1016/j.jvscit.2023.101139

**Published:** 2023-03-04

**Authors:** Ahmed K. Ghamraoui, Jake Forman, John Ricotta, Joseph J. Ricotta

**Affiliations:** a Charles E. Schmidt College of Medicine, Florida Atlantic University, Boca Raton, FL; b Department of Vascular Surgery, Delray Medical Center, Delray Beach, FL; c Tenet Healthcare, Delray Beach, FL

**Keywords:** Arteriotomy closure device, Femoral closure, Office-based laboratory, Peripheral angiogram, Peripheral angiography, Vascular closure device

## Abstract

**Objective:**

Manual compression remains the gold standard for achieving hemostasis for percutaneous common femoral artery access. However, it requires prolonged bedrest and 20 to 30 minutes or more of compression for hemostasis. Current arterial closure devices have emerged in recent years, but patients still require prolonged bedrest and time to ambulation and discharge, and these devices are associated with significant access device complications, including hematoma, retroperitoneal bleeding, transfusion requirement, pseudoaneurysm, arteriovenous fistula, and arterial thrombosis. A novel femoral access closure device, the CELT ACD (Vasorum Ltd, Dublin, Ireland), has been previously shown to reduce these complication rates and allow rapid hemostasis, require little or no bedrest, and shortened time to ambulation and discharge. This is especially advantageous in the outpatient setting. We report our initial experience with this device.

**Methods:**

A prospective single-center single-arm study was performed in an office-based laboratory setting to assess the safety and efficacy of the CELT ACD closure device. Patients underwent diagnostic and therapeutic peripheral arterial procedures from retrograde or antegrade common femoral artery access. Primary endpoints include device deployment success, time to hemostasis, and major or minor complications. Secondary endpoints include time to ambulation and time to discharge. Major complications were defined as bleeding requiring hospitalization or blood transfusion, device embolization, pseudoaneurysm formation, and limb ischemia. Minor complications were defined as bleeding not requiring hospitalization/blood transfusion, device malfunction, and access site infection.

**Results:**

A total of 442 patients were enrolled with common femoral access only. Median age was 78 years (range, 48-91 years), and 64% were male. Heparin was given in all cases, with median heparin dose of 6000 units (range, 3000-10,000 units). Protamine reversal was used in 10 cases due to minor soft tissue bleeding. Average time to hemostasis was 12.1 seconds (±13.2 seconds), time to ambulation was 17.1 minutes (±5.2 minutes), and time to discharge was 31.7 minutes (±8.9 minutes). All devices (100%) were deployed successfully. No major complications occurred (0%). Ten minor complications (2.3%) occurred; all were minor soft tissue bleeding from the access site that resolved with protamine reversal of heparin and manual compression.

**Conclusions:**

The CELT ACD closure device is safe and easily deployed with a very low complication rate, and significantly reduces time to hemostasis, ambulation, and discharge in patients undergoing peripheral arterial intervention from a common femoral artery approach in the office-based laboratory setting. This is a promising device that deserves further evaluation.

Manual compression remains the gold standard for achieving hemostasis for percutaneous common femoral artery access. However, it requires prolonged (4-6 hours) bedrest and 20 to 30 minutes or more of compression for hemostasis. Since their introduction in the early 1990s, many arterial closure devices have emerged, but they still require prolonged bedrest (≥2 hours) and are associated with significant access device complications, including hematoma, retroperitoneal bleeding, transfusion requirement, pseudoaneurysm, arteriovenous fistula, and arterial thrombosis. The CELT ACD (Vasorum Ltd, Dublin, Ireland), a novel femoral access closure device utilizing a stainless-steel clip, has been previously shown to reduce these complication rates and allow rapid hemostasis and time to ambulation and discharge.^1^^,^^2^ This is especially advantageous in the outpatient setting. We report our initial experience with this device.

## Methods

The study was approved by the local Institutional Review Board. Data was collected prospectively for patients undergoing diagnostic and therapeutic peripheral arterial procedures from retrograde or antegrade common femoral artery access in the office-based laboratory, consecutively between November 1, 2021, and October 1, 2022. Informed consent was obtained from all patients prior to intervention.

### Study criteria

Study inclusion criteria were (1) patients in the office-based laboratory setting, (2) undergoing diagnostic or therapeutic lower extremity peripheral arterial endovascular procedure from a retrograde or antegrade common femoral approach, using (3) 5 Fr, 6 Fr, or 7 Fr access site, with (4) minimum common femoral artery diameter of 4.8 mm. Exclusion criteria were (1) common femoral artery diameter less than 4.8 mm, (2) unable to visualize common femoral artery with ultrasound.

### CELT ACD technique

All access site closures using the CELT ACD were performed under ultrasound guidance. Following completion of the peripheral angiographic procedure, the tip of the CELT ACD vascular closure device was inserted through the valve of the introducer sheath, and the device was advanced and latched onto the sheath per manufacturer guidelines. The common femoral artery was then viewed under ultrasound on a longitudinal axis, and the device and introducer sheath were withdrawn together until the distal end of the pre-attached implant was visualized between the arteriotomy site and back wall of the common femoral artery. The distal wings of the implant were then deployed by rotating the handle clockwise, with wing opening confirmed with ultrasound. The device and sheath were then further withdrawn until resistance was felt and the distal wings were visualized flush at the luminal aspect of the arteriotomy. The device and sheath were then raised until perpendicular with the patient, and handle rotated counterclockwise to deploy the proximal wings, which was also confirmed with ultrasound. The implant was then ejected from the delivery system and a still fluoroscopic image taken to ensure the implant was present at the deployment site stabilized in the arterial wall (Fig).

**Fig:**
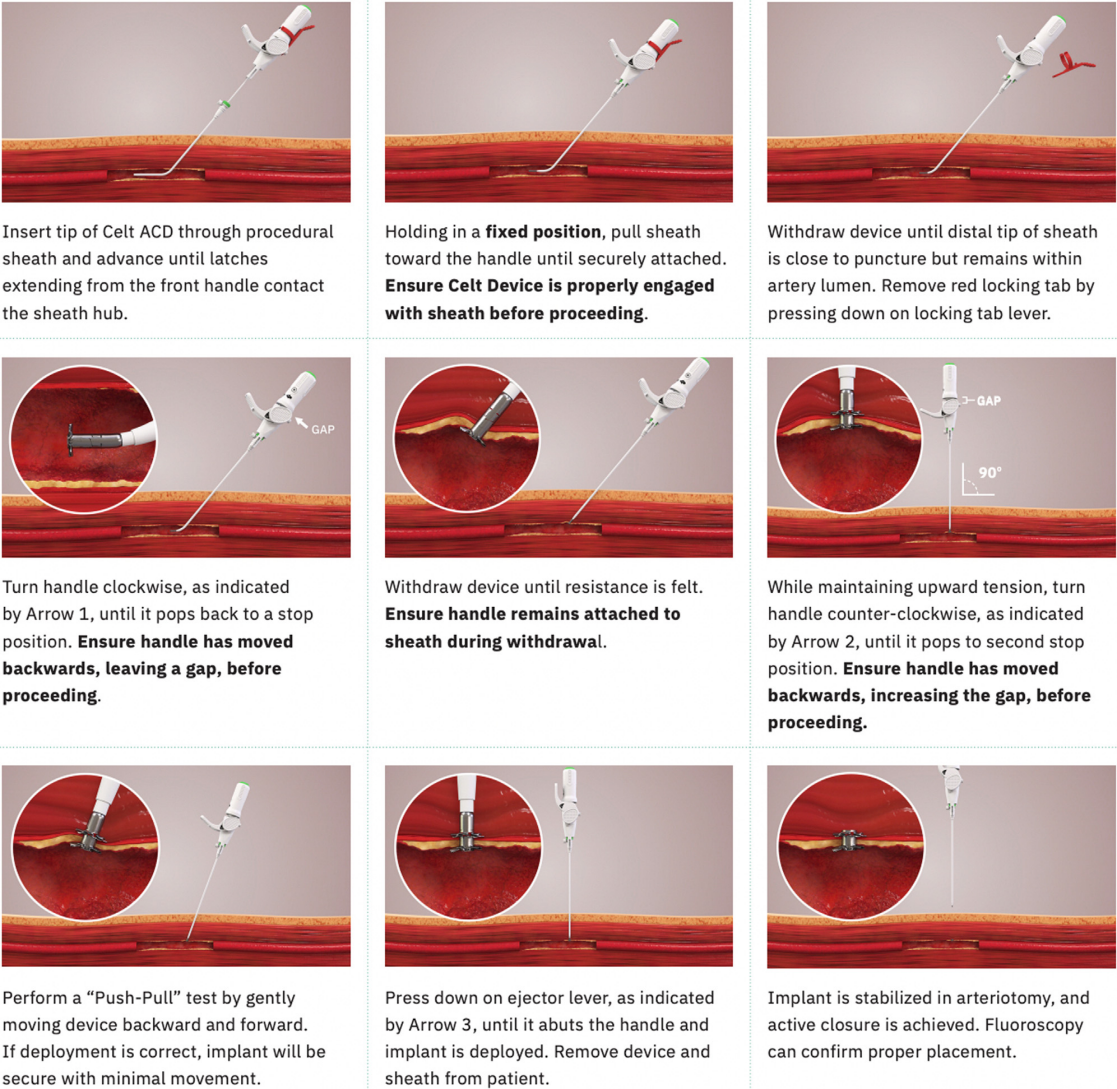
Deployment mechanism of the CELT ACD femoral arteriotomy closure device

### Endpoint definitions

Primary endpoints include device deployment success, time to hemostasis, and major or minor complication rates. Secondary endpoints include time to ambulation and time to discharge. Major complications were defined as bleeding requiring hospitalization or blood transfusion, device embolization, pseudoaneurysm formation, and limb ischemia. Minor complications were defined as bleeding not requiring hospitalization/blood transfusion, device malfunction, and access site infection.

## Results

A total of 450 peripheral angiograms were consecutively performed via common femoral access during the designated period, of which 442 were included in the study. Eight patients had common femoral arteries that were unable to be adequately visualized in a longitudinal axis on ultrasound due to excessive depth or scar tissue and thus excluded. Twenty-seven of the included angiograms were performed via an antegrade common femoral approach. In the study group, patients had an average age of 78 years (range, 48-91 years), with an average body mass index of 30 kg/m^2^ (range, 17-40 kg/m^2^), and 283 (64%) were male. The mean common femoral artery diameter was 7.7 ± 1.2 mm, with a median sheath size of 6 Fr. Table I summarizes the patient demographics and procedural variables. The eight patients that were excluded underwent common femoral access closure using MynxGrip (AccessClosure Inc, Santa Clara, CA) vascular closure device.

**Table I:** Patient demographics/procedural variables

	(N = 442*)*
Age, y	78 (48-91)
Sex	
Male	283 (64)
Female	159 (36)
BMI, kg/m^2^	30 (17-40)
CFA diameter, mm	7.7 ± 1.2
Antegrade CFA access	27 (6.1)
Retrograde CFA access	415 (93.9)
Heparin dose, units	6000 (4000-10,000)
Protamine given	10 (2.3)

*BMI*, Body mass index; *CFA*, common femoral artery.

Age, BMI, and heparin data presented as median with range; CFA diameter presented as mean ± standard deviation; other data is presented as number (%).

Heparin was given in all cases, with median heparin dose of 6000 units (range, 3000-10,000 units). Average time to hemostasis was 12.1 ± 13.2 seconds, time to ambulation was 17.1 ± 5.2 minutes, and time to discharge was 31.7 ± 8.9 minutes. Technical success was achieved in all cases, with no incidence of a major complication immediately following the procedure through 1-month follow-up. Ten minor complications (2.3%) occurred; all were minor soft tissue bleeding from access site that resolved with protamine reversal of heparin and manual compression (Table II).

**Table II:** Composite primary and secondary endpoints

	(N = 442*)*
Device success	442 (100)
Major complication	0 (0)
Minor complication	10 (2.3)
Time to hemostasis, sec	12.1 ± 13.2
Time to ambulation, min	17.1 ± 5.2
Time to discharge, min	31.7 ± 9.0

Data presented as number (%) or as mean ± standard deviation.

## Discussion

The common femoral artery is the preferred access site for peripheral angiography; it is easily visualized with ultrasound guidance and allows adequate platform length for successful revascularization of varied lesions, both anatomically and in severity.^3^ However, femoral access complications occur in 1.4% to 3.7% of patients, and include hematoma, retroperitoneal bleeding, transfusion requirement, pseudoaneurysm, arteriovenous fistula, and arterial thrombosis.^4^, ^5^, ^6^ Additionally, obtaining hemostasis following common femoral artery access can be arduous, requiring 20 to 30 minutes of manual compression, and yet is still considered the gold standard of care. Since arterial closure devices were first introduced in the early 1990s, their market has grown exponentially, with an estimated $1 billion market value as of 2013.^7^ Despite the growth and variety of vascular closure devices, they can still require prolonged bedrest and are associated with increased complications when compared with manual compression, such as local infection, distal embolization, and arterial thrombosis.^7^^,^^8^

The CELT ACD is a novel femoral access closure device that has been previously shown to reduce complication rates and allow rapid hemostasis, while reducing time to ambulation and discharge.^1^^,^^2^ This study parallels the ones prior, confirming rapid hemostasis and short time to ambulation and discharge. A reduction in femoral access complication rates has been shown to have an impact on patient mortality, with prior database studies showing a four-fold increase in 30-day mortality in patients with severe access site complications (6.1%), and at 1 year, the highest mortality rates were in patients with an access site complication that required blood transfusion (12.1%).^8^

In addition to having lower complications rates, the CELT ACD also obviates the need for prolonged bed rest, improving patient comfort in the recovery period. Furthermore, the decreased complication rate and shorter recovery times have been shown in the hospital setting to allow for the use of fewer resources and shorter stays, leading to a reduction in overall cost.^9^ With a price set similar to other closure devices currently on the market, this could translate to a significantly decreased cost in the outpatient/office-based laboratory setting. This study showed an average time to ambulation of 17.1 minutes and time to discharge of 31.7 minutes, with both these times limited by institutional policies for discharge after moderate sedation and nursing availability to assist patients. Additionally, it should be noted that, for the first 130 patients, an arbitrary minimum 20-second manual compression time was used. This was eliminated for the last 312 patients of the study, with hemostasis effectively achieved with zero seconds of manual compression time. This minimum 20-second manual compression time was felt to be part of the learning curve for the device. Prior to initial data collection for this study, the authors had used the device for 2 weeks and a total of approximately 20 patients; thus, the learning curve for actual device use is short. However, we found that physician comfort with abandoning additional manual compression lengthened this curve.

A potential negative for the CELT ACD is having a foreign body left behind. In our experience, we have had no discernable adverse effects stem from this; however, long-term effects are yet to be seen.

## Conclusions

The CELT ACD significantly reduces time to hemostasis while mitigating the need for prolonged bedrest and with minimal complications. It reduces time to ambulation and discharge in patients undergoing peripheral arterial intervention from a common femoral artery approach in the office-based laboratory setting. This is a promising device that deserves further evaluation.
